# Availability of Home sleep apnea test equipment LS-140 on a comparison with Polysomnography

**DOI:** 10.20407/fmj.2020-014

**Published:** 2021-03-20

**Authors:** Yuki Mieno, Masamichi Hayashi, Mariko Hirochi, Aki Ikeda, Hisashi Kako, Takuma Ina, Yuri Maeda, Shingo Maeda, Takahiro Inoue, Tomohide Souma, Toshikazu Watanabe, Tomoya Horiguchi, Yusuke Gotoh, Yoshikazu Niwa, Kumiko Yamatsuta, Sayako Morikawa, Yosuke Sakakibara, Takuya Okamura, Sakurako Uozu, Yasuhiro Goto, Sumito Isogai, Shiho Fujita, Junichi Fukumoto, Nami Hosoda, Kazuyoshi Imaizumi

**Affiliations:** 1 Department of Respiratory Medicine Ⅰ, Fujita Health University, School of Medicine, Toyoake, Aichi, Japan; 2 Fujita Health University Okazaki Medical Center, Okazaki, Aichi, Japan; 3 Fujita Health University Clinical Laboratory Center, Toyoake, Aichi, Japan

**Keywords:** Sleep apnea syndrome, Portable sleep monitoring, Polysomnography

## Abstract

**Objective:**

The prevalence of obstructive sleep apnea (OSA) in Japan is 9% among males and 3% among females. Up to 2.5 million patients are estimated to suffer from the disease, but limited number of facilities are capable of carrying out polysomnography (PSG), leaving more than 80% of these individuals are undiagnosed. In recent years, the development of new portable sleep monitoring (PMs) devices has been remarkable. We evaluate the correlation between the results of the LS-140 PMs device (Fukuda Denshi Tech Co. Ltd.), released in 2017, and those of PSG.

**Methods:**

We obtained contemporaneous data from the same patients by equipping 58 patients with PMs (LS-140) devices while they underwent PSG. Our primary outcome was Case 2 of the intraclass correlation coefficient (ICC), i.e., the ICC (2.1). And we used a Bland-Altman analysis to compare the apnea-hypopnea index (AHI) given by PSG and the respiratory event index (REI) given by LS-140 and examined the sensitivity and specificity of the REI relative to the AHI in the diagnosis of OSA. We also carried out the same comparison but in terms of the presence or absence of periodic limb movements (PLMs).

**Results:**

The ICC (2.1) between The REI and the AHI was 0.944, a rather high value (p<0.0001). The mean difference between AHI and REI values was –3.6 (p<0.0001), indicating a negative fixed bias. Sensitivity may decrease in groups with PLMs.

**Conclusion:**

The REI and the AHI are highly correlated, giving LS-140 sufficient diagnostic sensitivity and specificity to screen for OSA.

## Introduction

The gold standard for diagnosis of obstructive sleep apnea (OSA) is all-night polysomnography (PSG). The American Academy of Sleep Medicine (AASM) has declared PSG to be a standard monitor (Type 1: requiring recording on at least seven channels, including electroencephalogram, electrooculogram, chin electromyogram, electrocardiogram, respiratory airflow, respiratory movement, and oxygen saturation, constant monitoring by a specialized technician, and performance under supervision). However, because it requires specialized facilities and experienced technicians, the number of facilities capable of administering PSG are limited, and multiple individuals cannot easily be monitored at once.

OSA involves repeated partial or complete obstruction of the airway at the oropharynx during sleep, causing intermittent hypoxemia and sleep disruption, which in turn can cause arteriosclerosis, dysglycemia, and dyslipidemia, leading to coronary artery disease and cerebrovascular disease.^1^ Frequent apnea and hypopnea during sleep cause patients to present with sleep disorders, giving rise to excessive drowsiness and fatigue during the day, and decreased attention, causing traffic and workplace accidents; which researchers have noted as a clear societal danger.^2^ Reports indicate that the prevalence of OSA among Japanese people is approximately 9% in males^3^ and 3% in females.^4^ An estimated 9 million individuals in Japan have an apnea hypopnea index (AHI) of 15 or more per hour,^5^ but the number of patients on Continuously positive airway pressure (CPAP) treatment nationwide is less than 500,000,^6^ leading many to believe that most individuals with OSA are undiagnosed.^7^

In recent years, in an effort to enable unsupervised sleep testing at home, the number of channels required for testing and guidelines for using portable sleep monitoring (PMs) devices, which patients can use in their own homes, have been reduced. In order for these devices to gain widespread acceptance, the following are necessary: ① demonstration that respiratory-event-based diagnosis is sufficiently sensitive and specific, ② ease of use, such that patients can use the device without omission or error, and ③ the ability to put obtained data through the same manual analyses that PSG data undergoes.

Because of the difficulty associated with using them, most PMs devices do not include Electroencephalogram (EEG) sensors, hence, they cannot distinguish sleep. Normally, the AHI is calculated by finding the total number of apnea and hypopnea events during sleep and dividing it by total sleep time (TST). However, due to lack of EEG sensors, PMs devices use total recording time (TRT) instead, which can easily lead to an overestimation of sleep time. Respiratory event index (REI) does not always include hypopnea (recognized as arousal via EEG activity), and instead calculates the number of respiratory events per hour, which can easily lead to it exhibiting lower values than those given by PSG.^8^ Thus, PMs devices are not appropriate for the diagnosis of sleep disorders that can cause arousal via means other than respiratory events (such as insomnia, central sleep apnea, alveolar hypoventilation syndrome, periodic limb movement [PLM], etc.), nor are they recommended for the diagnosis of OSA in patients with these conditions.^9^ However, the effect of PLMs on PMs evaluation of respiratory events is still unclear.

In terms of the analysis of PMs data, the automatic analysis software built into most devices is still quite inaccurate,^10,11^ and analysis by a trained technician is recommended.^12^ Expert analyses can estimate arousal/sleep on the basis of pulse variations, changes in percutaneous oxygen saturation of peripheral arteries (SpO_2_), disturbances in respiratory waveforms, and changes in breath counts, enabling the subtraction of arousal time from the device’s TRT. In all, these adjustments can improve the extent to which the PMs results approximate the PSG results.^13^

Social awareness of sleep-disordered breathing has increased, and in the recent years, use of PMs devices in clinical settings has continued to rise. Even today, efforts to develop more accurate, useful, and convenient machines are underway. A judgment manual released by AASM in 2007 has standardized the scoring of collected data by technicians.

## Method

### Objective

In this study, we used the PulSleep LS-140 (Fukuda Denshi Co., Ltd.), a new, Type 3 PMs device released in 2017 (hereafter, LS-140). With the possibility of its use in screening for diagnosis and treatment in mind, we evaluated the correlation between LS-140 results and concurrently running PSG results and examined the accuracy and limitations of the device.

### Patients

This was a single-center, prospective study carried out at the Fujita Health University School of Medicine. Patients who visited the hospital affiliated with our university between November 2018 and October 2019 and who were slated to receive PSG testing due to the possibility of diagnosis of OSA were asked to participate in this study. We received written consent for participation from 60 individuals. Minors, individuals without sufficient reasoning ability, and individuals who were not conscious or aware were excluded from the scope of this study. Two individuals later withdrew their consent. Thus, a total of 58 patients were included in this study.

### Measurement

All patients underwent PSG testing at Fujita Health University Hospital. During the testing, the patients were also fitted with PMs device (LS-140) for enabling the contemporaneous collection of data from both devices and from the same patients. Once the testing had started, no interruptions or corrections were made, even if the sensors fell off. The PSG apparatus used was the Somnoscreen (SOMNOmedics GmbH, Germany), and testing was carried out in the Sleep Disorder Testing Room of the Fujita Health University Hospital. Using electrodes affixed to the surface of the body, we collected the following data: electroencephalogram, electrooculogram, chin electromyogram, tibialis anterior electromyogram, electrocardiogram. We also measured the air exhaled through patients’ nasal and oral cavities using temperature and pressure sensors. An inductance sensor with a strain gauge was used to measure the ventilation motion of the chest and abdomen. A pulse oximeter was used to measure SpO_2_. Postural measurements were taken using accelerometers fastened to the thorax and abdomen via belts. The LS-140 measured respiratory airflow (using a nasal cannula pressure sensor), respiratory effort (using a piezoelectric sensor), SpO_2_ and pulse (using a pulse oximeter), and posture (using an accelerometer). In order to ensure that the PSG and LS-140 received the same information for respiratory evaluation, the nasal pressure cannula was divided in two, and airflow was diverted into each of the two devices for measurement. All recorded data was manually analyzed by a single experienced technician, and judgment of apnea/hypopnea and sleep staging was done in accordance with Ver. 2.5 of The AASM Manual for the Scoring of Sleep and Associated Events for the Use of Unattended Portable Monitors in the Diagnosis of Obstructive Sleep Apnea in Adult Patients.^9^ A sustained (10 sec or more) ≥90% reduction in the signal amplitude of the lower nasal cavity temperature sensor of the PSG device was judged to be apnea. Similarly, a concurrent ≥30% reduction in the signal amplitude of the lower nasal cavity pressure sensor and a ≥3% decrease in SpO_2_, or an arousal response, was judged to be hypopnea. The LS-140 monitored nasal cavity airflow using a pressure sensor; a sustained (10 sec or more) ≥90% reduction in its signal amplitude was judged to be apnea, whereas a concurrent ≥30% reduction in signal amplitude and a ≥3% decrease in SpO_2_ was judged to be hypopnea. When analyzing LS-140 results, times of clear arousal were judged via disturbances in body movements and respiratory waveforms and variations in breath counts. This time was subtracted from recording time to yield the TRT value used in our analyses.

### Statistical Analysis

The primary outcome was Case 2 of the intraclass correlation coefficient (ICC), or ICC (2.1), which was used to compare the values measured by PSG and LS-140. Evaluation of confidence factors was done in accordance with Landis criteria.

As the secondary outcome, PSG and LS-140 measured values were compared using a paired t-test. Bland-Altman analysis was used to evaluate the correspondence between the AHI given by PSG and the REI given by LS-140. Using AHI values of 5 per hour, 15 per hour, and 30 per hour as diagnostic cutoffs, we created an REI receiver operating characteristic curve (ROC curve) to calculate and compare REI sensitivity and specificity. Finally, we separated patients into two groups based on a PLM index cutoff value of ≥15 per hour. The ICC between PSG and LS-140 measured values and the sensitivity and specificity of REI for the diagnostic cutoffs of AHIs of 5 per hour, 15 per hour, and 30 per hour were then evaluated for each group.

Statistical analyses were carried out in JMP ver 14 (Japanese ver.) for PC (SAS Institute Inc, Tokyo) and SPSS Statistics 21 (Stats Guild Inc, Chiba).

This study was approved by the Fujita Health University Research Ethics Committee under the title “A comparison study of the LS-140 PMs device and all-night polysomnography (approval no. HM18-048).”

## Results

Patient characteristics (Table 1): 58 patients participated in this study (48 males, 10 females). Median age (quartiles) was 54 (43.8–63) years, median body mass index (quartiles) was 25 (22.9–29.2) kg/m^2^, median AHI (quartiles) was 29.1 (20–42.4) per hour, with 3 patients in the normal category (AHI <5 per hour), 6 in the mild category (5≤AHI<15 per hour), 22 in the moderate category (15≤AHI<30 per hour), and 27 in the severe category (AHI ≥30 per hour). Eighteen of these patients exhibited a PLM index of ≥15 per hour. The LS-140 oxygen saturation sensor of one of the 58 patients studied fell off during testing, causing a loss of data.

Primary outcome: Comparison of PSG and LS-140 measured values (Table 2).

The ICC (2.1) between the AHI measured by PSG and the REI measured by LS-140 was 0.944 (almost perfect), a very high value (p<0.0001). The apnea index (AI), mixed apnea index (MAI), and 3% oxygen desaturation index (3% ODI) all exhibited confidence factors of 0.61 (substantial) or more (p<0.0001). No significant correlation was observed for central apnea index (CAI) or maximum duration of apnea. The correlation between TST as measured by the PSG and TRT as measured by LS-140 was fair, at 0.341.

**Figure 1:**
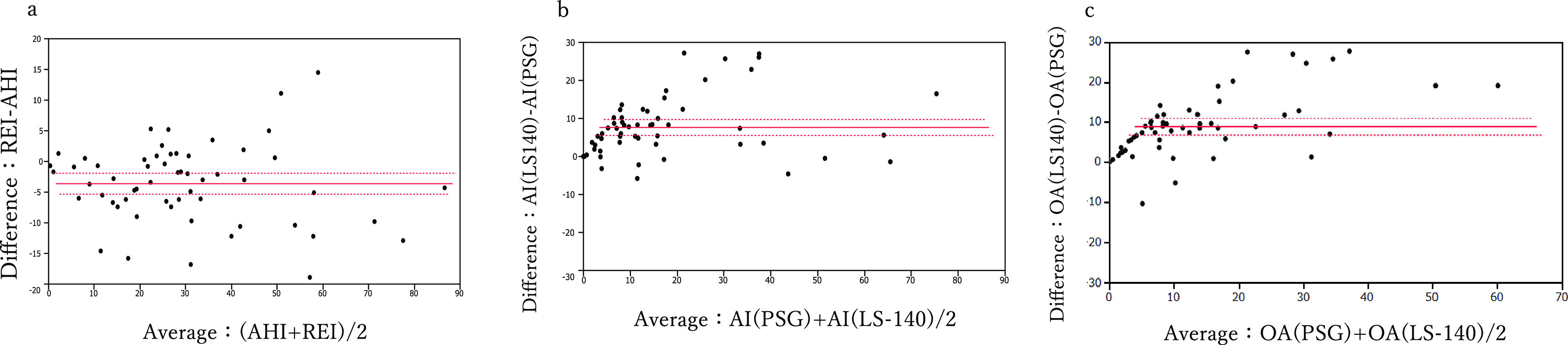
Brand-Altman limits of agreement and mean differences between three PSG variables and REI of LS-140. a: PSG variable: AHI mean difference: –3.6 (p<0.0001), The 95% confidence interval: –5.34~–1.93, Limits of Agreement: 3.41 b: PSG variable: AI mean difference: 7.62 (p<0.0001), The 95% confidence interval: 5.53~9.71, Limits of Agreement: 4.18 c: PSG variable: OA mean difference: 8.98 (p<0.0001), The 95% confidence interval: 6.91~11.05, Limits of Agreement: 4.14

Secondary outcome: LS-140 measured significantly higher values for AI, Obstructive Apnea(OA), 3% ODI, and % time of SpO_2_ <90%, and significantly lower values for HI, REI, and Lowest O_2_ (Table 1). As for the Bland-Altman analysis of AHI and REI, the mean difference between the two metrics with AHI as the standard was –3.6 (p<0.0001), with a 95% confidence interval of –5.34– –1.93 (limit of agreement 3.41), indicating that while REI has a negative fixed bias relative to AHI, the values produced by both scales were comparable. The mean difference for AI with PSG as the standard was 7.62 (p<0.0001), with a 95% confidence interval of 5.53–9.71 (limit of agreement 4.18); therefore, a positive fixed bias for LS-140 AI relative to PSG AI was observed. Similarly, the mean difference for OA with PSG as the standard was 8.97 (p<0.0001), with a 95% confidence interval of 6.91–11.05 (limit of agreement 4.14); therefore, a positive fixed bias for LS-140 OA relative to PSG OA was observed (Figure 1).

We used an ROC curve to calculate the AHI diagnostic performance of REI (Table 3). The areas under the ROC curve were as follows: 1.000 if AHI ≥5 per hour was taken as positive, 0.986 if AHI ≥15 per hour was taken as positive, and 0.962 if AHI ≥30 per hour was taken as positive. All of these were extremely high values. If an AHI ≥5 per hour was used as the cutoff, the sensitivity and specificity of REI were 0.964 and 1.000, respectively, whereas if an AHI ≥30 per hour was taken as the cutoff, REI sensitivity and specificity were 0.741, and 1.000, respectively.

The patient characteristics of the 18 patients in the PLMs and the 40 patients in the non-PLMs groups are given in Table 4. The 18 patients in the PLMs group (16 males, 2 females) were significantly older than those in the non-PLMs group (p=0.011) and tended to have higher PSG Lowest SpO_2_ values (p=0.04). No significant difference in AHI was observed (p=0.094). Their LS-140 Lowest SpO_2_ values were higher (p=0.024), and their 3% ODI values were lower (p=0.009).

The AHI and REI ICC (2.1) in the non-PLMs group was 0.945 (p<0.0001), and the AI ICC (2.1) was 0.911 (p<0.0001); both were quite high (almost perfect). However, the ICC (2.1) between TST and LS-140 TRT was 0.471 (p=0.001), indicating only a moderate level of confidence. The AHI and REI ICC (2.1) in the PLMs group was 0.927, almost perfect, but the AI ICC (2.1) was 0.233 (p=0.168), indicating no significant correlation. Similarly, no significant correlation was observed between TST and LS-140 TRT (Table 5).

The areas under the ROC curve for the PLMs group for various AHI positive cutoffs were as follows: AHI ≥5 per hour, 1.000; AHI ≥15 per hour, 0.946; AHI ≥30 per hour, 0.961. All of these were very high values. At a cutoff of AHI ≥5 per hour, REI sensitivity and specificity were 0.938 and 1.000, respectively, and at a cutoff of AHI ≥30 per hour, REI sensitivity and specificity were 0.429 and 0.909, respectively (Table 6).

The areas under the ROC curve for the non-PLMs group for various AHI positive cutoffs were as follows: AHI ≥5 per hour, 1.000; AHI ≥15 per hour, 1.000; AHI ≥30 per hour, 0.976. All of these were very high values. At a cutoff of AHI ≥5 per hour, REI sensitivity and specificity were 0.974 and 1.000, respectively, and at a cutoff of AHI ≥30 per hour, REI sensitivity and specificity were 0.948 and 1.000, respectively.

## Discussion

With the increasing use of PMs devices, many studies are being performed to measure their accuracy and utility.^14–16^ A meta-analysis of PSG and Type 3 PMs devices published in 2014 stated that if Type 3 PMs devices (capable of recording the smallest number of channels, including airflow or respiratory movement, pulse rate or EKG, and oxygen saturation) are used in adults with high pretest probability and under supervision, and if their data is subsequently analyzed by specialized technicians according to the AASM Ver. 2.5 manual, these devices are sufficiently reliable. However, in practical application, the number of channels measured varies from facility to facility, with 4-channel measurement used most commonly (69.5%), followed by 3-channel measurement (10.1%).^17^ The LS-140 used in this study uses pressure sensors to record respiratory airflow, mask pressure, and tracheal sound data, a piezoelectric sensor and accelerometers to record respiratory effort, postural, and body movement data, and a pulse oximeter to record SpO_2_ and pulse rate data. Here, we collected 5-channel measurements: respiratory airflow, respiratory effort, posture, SpO_2_, and pulse. As judged by PSG data, 84% of our 58 patients had moderate to severe OSA. They were checked at an outpatient facility, where if they had a possible diagnosis of sleep apnea syndrome, only then they received PSG testing. Thus, their pretest probability was likely higher than that of the average population. None of the patients exhibited central apnea, including Cheyne-Stokes breathing; obstructive-dominant apnea and hypopnea were predominant.

PSG-measured AHI and LS-140-measured REI exhibited an ICC (2.1) of 0.944 (almost perfect), a high value (p<0.0001, Table 2). In a randomized crossover trial published in 2014,^17^ the ICC between PSG and in-facility PMs was 0.79 (95% confidence interval 0.67–0.86); our results exceeded this value.

In this study, the intraclass correlations of respiratory events were quite high, but the intraclass correlation between PSG-measured TST and LS-140-measured TRT was only 0.341 (fair). We believe that the absence of any significant difference between TST and TRT mean differences (Table 1), contributed to the high correlation between REI and AHI. When PMs devices are used in clinical settings, technicians can subtract the times during which patients were moving, their breathing patterns were disturbed, and other clear periods of arousal from the TRT tracked by the device. This is believed to improve the extent to which the PMs results approximate the PSG results.^10^ However, in this study, the low ICC between TST and TRT indicates that the two values are subject to a significant degree of variance, and that issues with inter-rater reliability remain.

In comparison to PSG, the LS-140 exhibited a positive fixed bias for AI and OA (Figure 1). In this study, the nasal pressure cannula affixed to patients at the start of testing was divided into two streams such that both the PSG and the LS-140 would be provided with equivalent air pressure. We believe that the constant fixed bias observed for AI and OA may be due to certain particularities of the LS-140 device itself.

Regardless of whether an AHI cutoff of ≥5 per hour, ≥15 per hour, or ≥30 per hour was used, the area under the ROC curve never fell below 0.96, indicating very high diagnostic performance. Relative to AHI, the sensitivities of REI ranged from 74.1–96.4%, depending on the specific diagnostic cutoff used, whereas all specificities were 100%. In a previously published meta-analysis,^17^ the mean sensitivity (95% confidence interval) of PMs to diagnose an in-facility PSG-measured AHI ≥5 per hour was 97 (92–99)%, whereas the median specificity (quartiles) was 93 (89–96)%. Our results for sensitivity were nearly equivalent to this result, and our specificity surpassed this result.

Previous research has pointed out that because it cannot evaluate limb movement, PMs device cannot evaluate sleep disruption due to PLMs.^18^ Here, the median value (quartiles) for the PLM index (times/hour) across all patients was 1 (0–28.7); 18 out of 58 patients (31%) exhibited a PLM index ≥15 per hour. In the Wisconsin Sleep Cohort study, the prevalence of a PLM index ≥15 per hour in the general population (median age 48 years) was 25.3%; this value increased in higher age brackets, ultimately reaching 34%.^19,20^ The age bracket of patients in this study was around 54 (43.8–63); our results therefore do not contradict previous findings.

In PLMs group, REI sensitivity when using a cutoff of AHI ≥30 per hour fell greatly, to 42.9%. The tendency of PMs (which lack EEG functionality) to underestimate respiratory events worsens as post-sleep-onset arousal increases,^8^ which may have led to a decline in sensitivity in the PLMs group. Similarly, the positive likelihood ratios of REI were all ∞, regardless of which diagnostic cutoff was used, whereas negative likelihood ratios ranged from 0.259–0.555 depending on the diagnostic cutoff used. This latter set of results was slightly poorer than the mean value of 0.03 (0.01–0.08) given in the meta-analysis. While the negative predictive value across all patients was 81.6%, it was 90.9% in the non-PLMs group and 73.3% in the PLMs group; thereby, showing a clear difference. Thus, we believe that the performance of the device in the PLMs group affected its performance across all patients studied here, and that care must be taken when using PMs results to exclude the possibility of a severe condition.

Bland-Altman analysis of AHI and REI revealed a mean difference of –3.6 (p<0.0001), indicating a negative fixed bias (Figure 1). We believe this reflects the general tendency of PMs to underestimate respiratory events relative to PSG. Because the limit of agreement was 3.4, both scales correspond with one another, and the presence or absence of PLMs did not cause large differences. Our scatterplots show right-facing fan-like shapes, suggesting that in comparison to PSG values, PMs values are subject to larger errors as measured values increase. This may be due to the fact that the sensitivity of REI in the PLMs group fell considerably for a cutoff of AHI ≥30 per hour. REI sensitivity to AHI was highest when a cutoff of AHI ≥5 per hour was used (96.4%). We believed the fact that this value fell to 74.1% when a cutoff of AHI ≥30 per hour was used and was reflective of this increase in error. The correlation coefficient for REI-AHI difference with AHI was –0.2390 (95% confidence interval –0.04684–0.0205), with a p-value of 0.0708; thus, no significant correlation between these two values was found (Supplementary Materials).

The effect of the presence or absence of PLMs on PMs parameters remains unclear, and even the general trends of this effect are unknown.

### Limitations

In this study, a technician fitted patients with PMs device sensors; this is a different situation than what patients would experience when fitting themselves with sensors at home. Further, because this was not a double-blind trial, the possibility of observer bias cannot be eliminated, limiting the external validity of these results.

### Conclusion

AHI, as measured by PSG, and REI, as measured by the LS-140, were very highly correlated, and the latter possesses sufficient diagnostic sensitivity and specificity for the diagnosis of respiratory events. However, it is possible that PMs measurements may be greatly affected by the presence of PLMs in patients that experience them; because the prevalence of significant PLMs is around 25% in the general population, caution must be exercised when interpreting PMs results. LS-140 requires only three sensors: a nasal pressure sensor, a fingertip oxygen saturation sensor, and a belted posture sensor. Thus, if given an explanation by a technician and provided with illustrated instructions, patients should easily be able to fit the sensors themselves at home. Out of 58 patients studied, only one instance of sensor removal during sleep occurred in this study. Based on the above, we conclude that the LS-140 possesses sufficient functionality for use as an at-home, unsupervised screening tool for OSA.

## Supplementary Material

Supplementary Figure

PDF-Japanese

## Figures and Tables

**Table1:** Patient characteristics

			PSG	LS-140	p
			n=58		
Gender		%(male)	82.8	
Age		years old	54.0 (43.8–63)		
Body mass index		kg/m^2^	25.0 (22.9–29.2)		
Epworth sleepiness scale			7.0 (5.0–9.3)		
Apnea Index		events/hour	7.8 (2.1–15.2)	14.1 (8.8–35.3)	0.0001
Obstructive apnea		events/hour	5.8 (1.1–12.6)	13.4 (7.4–25.1)	<0.0001
Central apnea		events/hour	0 (0–0)	0 (0–0)	0.1231
Mixed apnea		events/hour	0.5 (0–3.7)	0.1 (0–1.7)	0.1338
Hypopnea Index		events/hour	18.6 (11.8–24.5)	5.4 (1.5–12.3)	<0.0001
AHI/REI		events/hour	29.1 (20–42.4)	26.1 (13.3–37.1)	<0.0001
AHI	<5 per hour	%	5.2		
	5–15 per hour	%	10.3		
	15–30 per hour	%	37.9		
	>30 per hour	%	46.6		
REI	<40 per hour	%		77.6	
	>40 per hour	%		22.4	
Maximum duration of apnea		seconds	49.0 (29.8–69.0)	82.0 (68.0–101.5)	0.184
3% oxygen desaturation index		times/hour	24.4 (13.7–36.7)	32.9 (16.1–45.4)	<0.0001
% Time of SpO_2_ <90%		%SPT	0.8 (0.1–2.2)	3.1 (0.8–6.4)	<0.0001
Lowest SpO_2_		%	83.5 (78.8–88.0)	79.0 (70.3–83.0)	<0.0001
Total Sleep Time		minutes	397.6 (350.9–456.6)	392.0 (330.5–437.5)	0.128
Sleep Efficiency		%SPT	80.0 (68.6–88.9)		
Wake after sleep onset		%SPT	19.0 (9.1–27.9)		
REM sleep		%TST	14.4 (11.1–18.4)		
Non-REM sleep 1st Stage		%TST	39.0 (29.5–52.6)		
Non-REM sleep 2nd Stage		%TST	42.8 (33.2–51.1)		
Non-REM sleep 3rd Stage		%TST	0.2 (0–5.1)		
Arousal Index		events/hour	34.3 (24.4–47.5)		
Periodic Limb Movement Index		times/hour	1.0 (0–28.7)		

AHI: Apnea Hypopnea Index, REI: Respiratory Event Index, REM: rapid eye movement, NREM: non rapid eye movement, PLM Index: Periodic Limb Movement Index.

**Table2:** Intraclass correlation between PSG and LS-140 measurements

	Interclass correlation	95% lower confidence limit	95% upper confidence limit	p
AHI and REI	0.944	0.907	0.967	<0.0001
Apnea Index	0.628	0.442	0.763	<0.0001
Hypopnea Index	0.541	0.328	0.701	<0.0001
Obstructive Apnea Index	0.566	0.362	0.718	<0.0001
Central Apnea Index	0.004	–0.253	0.260	0.4880
Mixed Apnea Index	0.801	0.685	0.877	<0.0001
Maximum duration of apnea	0.299	–0.190	0.587	0.0940
3% oxygen desaturation index	0.790	0.667	0.870	<0.0001
Lowest SpO_2_	0.943	0.902	0.966	<0.0001
TST and TRT	0.341	0.090	0.551	0.0040

PSG: Polysomnography, AHI: Apnea Hypopnea Index, REI: Respiratory Event Index, TST: Total sleep time,TRT: Total recording time.

**Table3:** Diagnostic performance of LS-140 taking different AHI obtained with PSG as the cutoff

	Sensitivity	Specificity	Positive predictive value	Negative predictive value	Positive likelihood ratio	Negative likelihood ratio	Area under the curve
AHI 5 per hour or more	0.964	1.000	1.000	0.600	∞	0.036	1.000
AHI 15 per hour or more	0.778	1.000	1.000	0.563	∞	0.143	0.986
AHI 30 per hour or more	0.741	1.000	1.000	0.816	∞	0.259	0.962

AHI: Apnea Hypopnea Index.

**Table4:** Patient characteristics according to PLMs group and non-PLMs group

			PLMs (n=18)	non-PLMs (n=40)
Gender		%(male)	88.9	80.0
Age		years old	67.5 (51.0–74.0)	53.0 (41.0–61.8)
Body mass index		kg/m^2^	25 (22.5–28.6)	25.1 (22.9–29.7)
Epworth sleepiness scale			7.0 (5.3–9.5)	7.0 (5.0–9.3)
PSG				
Apnea Index		per hour	8.9 (0.88–14.1)	7.6 (3.0–17.2)
Obstructive apnea		per hour	2.3 (0.6–11.3)	6.5 (1.6–15.5)
Central apnea		per hour	0 (0–0)	0 (0–0)
Mixed apnea		per hour	1.0 (0–4.6)	0.6 (0–3.4)
Hypopnea Index		per hour	13.8 (11.1–18.9)	20.1 (13.1–27.6)
Apnea Hypopnea Index		per hour	24.6 (17.7–32.7)	30 (20.4–46)
SDB severity	normal	%	11.1	2.5
	mild	%	11.1	10.0
	moderate	%	38.9	37.5
	severe	%	38.9	50.0
3% oxygen desaturation index		Times/hour	16.6 (12.0–30.1)	27 (14.4–43.3)
% Time of SpO_2_ <90%		%SPT	0.3 (0–1.8)	1.0 (0.2–3)
Lowest SpO_2_		%	86.0 (82.3–90.0)	82.0 (76.0–86.0)
Total Sleep Time		Minutes	377.0 (331.6–445.6)	402.2 (351.4–458.8)
Sleep Efficiency		%SPT	74.5 (68.2–88.3)	80.8 (71.1–90.7)
Wake after sleep onset		%SPT	22.9 (10.9–30.3)	18.2 (7.7–25.5)
REM sleep		%TST	13.5 (9.6–14.9)	15.2 (11.3–19.1)
Non-REM sleep 1st Stage		%TST	49.2 (36.4–54)	37.6 (26.5–47.5)
Non-REM sleep 2nd Stage		%TST	37.1 (25.6–46.7)	44.6 (35.4–53.4)
Non-REM sleep 3rd Stage		%TST	0 (0–5.0)	0.4 (0–5.3)
Arousal Index		Events/hour	42.3 (25–50.2)	33.8 (24.2–44.9)
Periodic Limb Movement Index		Times/hour	34.6 (30–51.5)	0 (0–1.2)
LS140				
Apnea Index		Events/hour	13.7 (3.6–26.8)	15.7 (10.7–35.1)
Obstructive apnea		Events/hour	11.7 (3.6–20.7)	14.7 (9.8–28.7)
Central apnea		Events/hour	0 (0–0)	0 (0–0)
Mixed apnea		Events/hour	0.4 (0–1.5)	0 (0–2.4)
Respiratory Event Index		Events/hour	21.2 (9.4–28.8)	28.1 (17.0–47.7)
3% oxygen desaturation index		Times/hour	25.8 (12.3–35.5)	34 (22.9–56.4)
Lowest SpO_2_		%	81.5 (78–88.3)	75.5 (69–81.3)
Total recording time		Minutes	401.5 (315.8–436.0)	387.0 (342.0–454.0)

PSG: Polysomnography, SDB: Sleep disorder index, REM: Rapid eye movement, PLMs: Periodic Limb Movements.

**Table5:** Intraclass correlation coefficients between markers obtained with PSG and LS-140 according to the presence of PLMs

	PLMs group					non-PLMs group			
	Intraclass correlation coefficient	95% lower confidence limit	95% upper confidence limit	p		intraclass correlation coefficient	95% lower confidence limit	95% upper confidence limit	p
AHI and REI	0.927	0.817	0.972	<0.0001		0.945	0.897	0.971	<0.0001
Apnea Index	0.233	–0.249	0.623	0.1680		0.911	0.837	0.952	<0.0001
Hypopnea Index	0.540	0.112	0.799	0.0090		0.519	0.248	0.716	<0.0001
Obstructive Apnea Index	0.171	–0.309	0.581	0.2420		0.830	0.702	0.907	<0.0001
Central Apnea Index	0.000	–0.456	0.456	0.5000		0.002	–0.306	0.310	0.4950
Mixed Apnea Index	0.688	0.338	0.871	0.0010		0.893	0.808	0.942	<0.0001
Maximum duration of apnea	0.050	–1.540	0.645	0.4590		0.472	–0.007	0.723	0.0260
Lowest SpO_2_	0.935	0.836	0.975	<0.0001		0.875	0.772	0.933	<0.0001
3% oxygen desaturation index	0.460	0.006	0.757	0.0240		0.830	0.700	0.907	<0.0001
TST and TRT	0.075	–0.394	0.513	0.3790		0.471	0.186	0.682	0.0010

AHI: Apnea Hypopnea Index, REI: Respiratory event index, TST: Total sleep time, TRT: Total recording time, PLMs: Periodic Limb Movements.

**Table6:** Diagnostic performance of LS-140 according to PLMs group and non-PLMs group

PLMs	Sensitivity	Specificity	Positive predictive value	Negative predictive value	Positive likelihood ratio	Negative likelihood ratio
AHI ≥5 per hour	0.938	1.000	1.000	0.667	∞	0.062
AHI ≥15 per hour	0.786	1.000	1.000	0.571	∞	0.214
AHI ≥30 per hour	0.429	0.909	1.000	0.733	∞	0.571

non-PLMs	Sensitivity	Specificity	Positive predictive value	Negative predictive value	Positive likelihood ratio	Negative likelihood ratio
AHI ≥5 per hour	0.974	1.000	1.000	0.500	∞	0.026
AHI ≥15 per hour	0.886	1.000	1.000	0.556	∞	0.114
AHI ≥30 per hour	0.948	1.000	1.000	0.909	∞	0.100

AHI: Apnea Hypopnea Index, PLMs: Periodic Limb Movements.
