# Supplementary figures and images for: Availability of Home sleep apnea test equipment LS-140 on a comparison with Polysomnography
Source: Fujita Med J. 2021 Mar 20;8(1):17–24. doi: 10.20407/fmj.2020-014 (PMC8874914; doi:10.20407/fmj.2020-014)

Correlation between AHI and REI-AHI difference

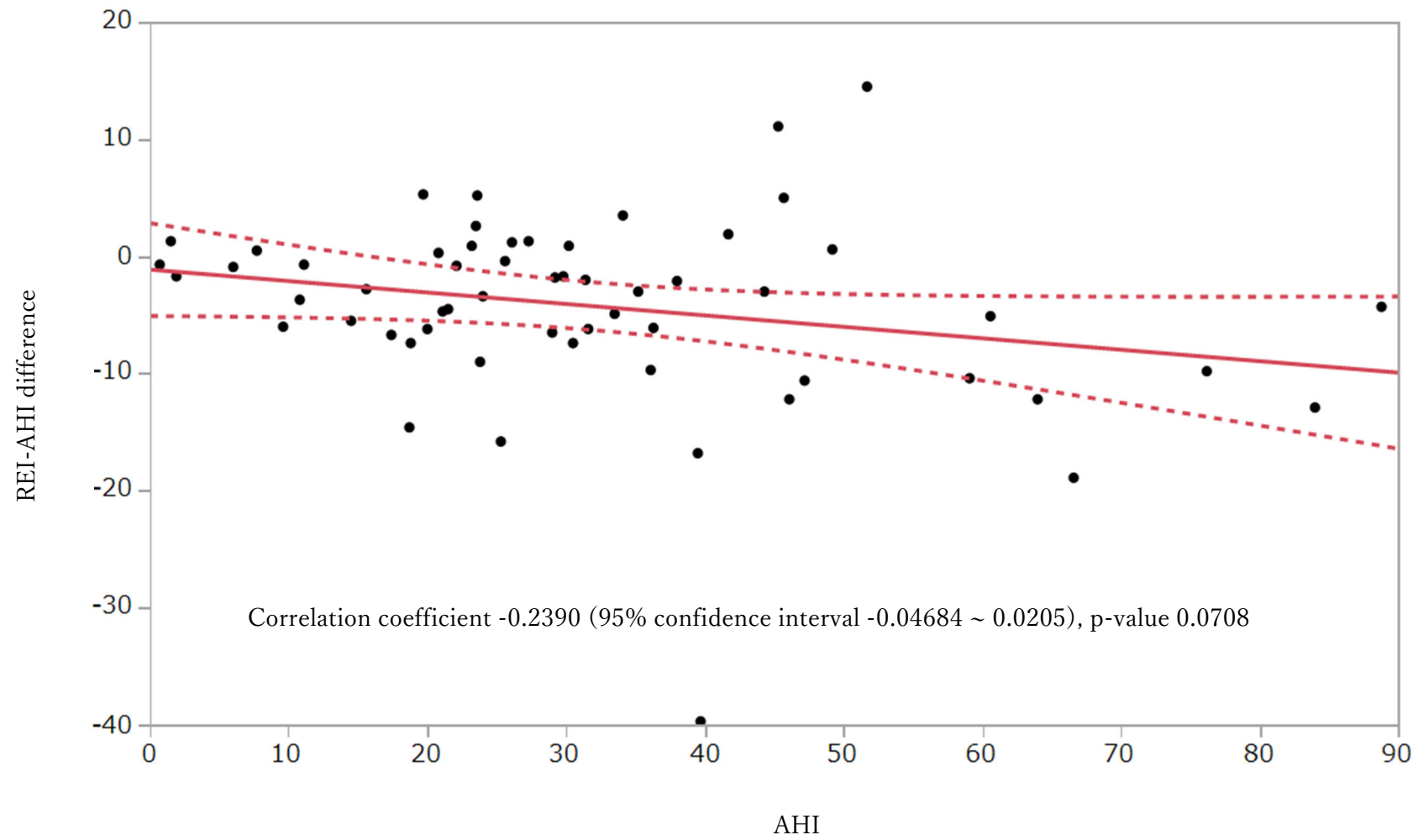

Supplement: Supplementary file 1 — Supplementary Figure [file fmj-8-017-s001.pdf]
