# Supplementary material for: Availability of Home sleep apnea test equipment LS-140 on a comparison with Polysomnography
Source: Fujita Med J. 2021 Mar 20;8(1):17–24. doi: 10.20407/fmj.2020-014 (PMC8874914; doi:10.20407/fmj.2020-014)
Supplement: Supplementary file 2 — PDF-Japanese [file fmj-8-017-s002.pdf]

アブストラクト

目的：日本人の閉塞性睡眠時無呼吸症候群(Obstructive Sleep Apnea：OSA)の有病率は、男性で約 9%、女性で約 3%とする報告がある。潜在的な患者数は 250 万人以上と推測されるが、終夜睡眠ポリグラフ検査(Polysomnography : PSG) を施行可能な施設は限られ、80%以上が未診断である。近年、OSA の診断における簡易モニター(Portable sleep monitoring：PMs)機器の利用方針が議論され、新機器の開発も著しい。2017 年発売の PMs 機器 LS-140(フクダライフテック社)と PSG との相関を評価する。

方法：OSA 疑い患者 58 名を対象に PSG 検査を実施する際、同時に PMs(LS140)を装着し、同一被検者による同一時刻のデータを取得した。主要評価項目として、PSG と LS140 の測定値を級内相関係数(Intraclass correlation coefficient: ICC)の Case2、ICC(2.1)を用いて比較した。副次評価項目として、PSG における無呼吸低呼吸指数（apnea hypopnea index：AHI）と LS140 の呼吸イベント指数(respiratory event index: REI)を Bland-Altman 分析を用いて評価し、AHI による OSA 診断に対する REI の感度と特異度を検討した。また、周期性四肢運動の有無別に同様の比較を行った。

結果：PSG 上の AHI と LS140 による呼吸イベント指数の ICC(2.1)は 0.944 と高値を示した(p<0.0001)。AHI と REI の差の平均値は-3.6(p<0.0001)と負の固定誤差を示した。周期性四肢運動を伴う群では感度が低下する可能性がある。

結論：LS140 上の呼吸イベント値と PSG における AHI との相関は非常に高く、OSA のスクリーニングにおいて十分な診断感度と特異度を有する。

キーワード

Sleep apnea syndrome, Portable sleep monitoring, Polysomnography.

序論

閉塞性睡眠時無呼吸症候群(Obstructive Sleep Apnea：OSA）の診断には終夜睡眠ポリグラフ検査(Polysomnography : PSG)がゴールドスタンダードである。アメリカ睡眠学会(The American Academy of Sleep Medicine: AASM)は PSG を標準的モニター(タイプ 1：脳波、眼電図、頤筋電図、心電図、呼吸気流、呼吸運動、酸素飽和度を含む最低 7 チャンネルの記録を行い、専門の技師の常駐、監視下で行う)と定めているが、専門施設と熟練した技師を要し、施行可能な施設は限られ、また多人数の検査の完遂は困難である。

OSA は睡眠中に中咽頭で気道の部分的または完全な閉塞が反復し、夜間の間欠的低酸素血症、睡眠の分断をきたす。間欠的低酸素は、動脈硬化、糖代謝異常、脂質代謝異常を惹起し、冠動脈疾患、脳血管障害を引き起こす[1]。患者は睡眠中の頻回な無呼吸、低呼吸によって睡眠障害を呈し、日中の過剰な眠気や倦怠感、注意力の低下を生じ、交通事故や労働災害を起こすなど社会的な危険性が指摘されている[2]。日本人の OSA の有病率は、男性で約 9%[3]、女性で約 3%[4]とする報告がある。日本には無呼吸低呼吸指数（apnea hypopnea index : AHI）15 回/時間以上の患者が 900 万人いると推測されている[5]が、国内で CPAP 治療を受けている患者数は 50 万人に満たず[6]、多くが未診断であると考えられている[7]。

近年、在宅・非監視下での睡眠評価を行うことを目的として、チャンネル数を減じ、患者自身が自宅で装着して使用する簡易モニター(Portable sleep monitoring：PMs)の利用方針が盛んに検討されるようになった。その運用には①呼吸イベントの診断に十分な感度と特異度を持つこと②取り扱いが簡便で、被験者自身が脱落や間違いを起こさず装着できること③取得したデータを PSG と同じくマニュアル解析にかけられることが必要となる。

在宅で行うモニターの多くは患者自身による装着の難しさから脳波センサーを欠き、睡眠と覚醒の区別がつけられないという問題がある。本来、無呼吸低呼吸指数（apnea hypopnea index : AHI）は 無呼吸数と低呼吸数の合計を全睡眠時間（Total Sleep time: TST）で割って算出されるが、脳波のない PMs では総記録時間（Total recording time: TRT）で割るため睡眠時間を過大評価しやすくなる。REI には脳波上の覚醒反応による低呼吸が含まれないこともあり、一時間当たりの呼吸イベント数が PSG での値よりも低くなりやすい[8]。よって、呼吸イベント以外での覚醒をきたす可能性がある睡眠障害(不眠症、中枢性無呼吸、肺胞低換気症候群、周期性四肢運動(Periodic Limb Movements:PLMs)など)の診断には PMs は適さず、また、これら疾患を持つ患者の OSA の診断にも推奨されていない[9]。しかし、PMs による呼吸イベント評価への PLMs の影響は未だ明らかではない。

PMs の解析においては、機器付属の自動解析はいまだ正確性が低く[10.11]、熟練した技師によるマニュアル解析が推奨されている[12]。習熟者による解析では脈拍や経皮的動脈血酸素飽和度(oxygen saturation of peripheral artery：SpO_2)の変動，呼吸波形の乱れ、呼吸数変動などから覚醒・睡眠をある程度推測することは可能であり、機器の総記録時間から明らかな覚醒時間をあらかじめ削除して評価することにより、PSG との近似性を改善するとされる[13]。

社会での睡眠呼吸障害の認知が進み、近年臨床現場において PMs の施行件数は増加の一途をたどっている。現在もさらなる正確性、有用性、利便性を追求した検査機器の開発が続いており、技師によるスコアリングも 2007 年に AASM が発行した判定マニュアルによる均一化が進んでいる。

方法

目的

この研究は、2017 年発売の新機器、タイプ 3 の PMs パルスリープ LS-140(フクダライフテック社)(以下 LS140)を用いて、診療におけるスクリーニングに使用することを念頭に置いて、現行の PSG との相関を評価し、正確性と限界を検討する。

対象

藤田医科大学で行った単施設、前向き研究である。2018 年 11 月から 2019 年 10 月までの期間に当大学病院を受診し、OSA の疑いにて PSG 検査を予定した患者に研究参加への協力を求め、60 名から書面による同意を得た。未成年、十分な判断力のない場合、意識の無い場合等、配慮が必要な場合は研究対象外とした。その後、2 名が同意を撤回したため、合計 58 名を対象として研究を行った。

測定方法

全被検者は藤田医科大学病院において PSG 検査を実施する際、同時に PMs(LS140)を装着し、同一被検者による同一時刻のデータを取得した。LS140 の装着は PSG 機器の装着と同時に技師が行い、検査開始後は測定終了まで脱落時にも修正は行わずに完遂した。PSG 機器は Somnoscreen(SOMNOmedics GmbH, Germany)を用い藤田医科大学病院の睡眠障害検査室で施行した。PSG と機器のセンサーは体表面に接着した電極より脳波、眼電図、頤筋電図、前脛骨筋筋電図、心電図、並びに鼻腔並びに口腔での気流を温熱、圧センサーを用いて測定した。胸部並びに腹部の換気運動測定にはストレインゲージによるインダクタンス方式のセンサーを使用した。また、パルスオキシメーターを用いて SpO_2 の測定を施行した。体位計測は胸部並びに腹部のベルトの加速度センサーにて記録した。LS140 は呼吸気流(鼻カニューレ式圧センサー)、呼吸努力(ピエゾセンサー)、パルスオキシメーターによる SpO_2 と脈拍数を、加速度センサーにより体位を測定した。PSG と LS140 が呼吸評価で同一の情報を得られる

よう、鼻圧カニューレを二分岐し両装置へ気流を分配し測定した。記録したデータはすべて熟練した技師一名によるマニュアル解析を行い、無呼吸と低呼吸、睡眠ステージの判定はAASM発布の睡眠及び随伴イベントの判定マニュアルver.2.5に従った[9]。PSG上で鼻腔下部の温熱センサーの信号振幅が10秒以上持続して90%以上低下した場合に無呼吸と判定し、同部位の圧センサーの信号振幅が 30%以上低下に加え SpO_2 が 3%以上低下、または覚醒反応を認めた場合に低呼吸と判定した。LS140 では鼻腔の気流を圧センサーで感知し、信号振幅がベースラインより 90%以上低下し 10 秒以上持続した場合を無呼吸と判定し、信号振幅が 30%以上の低下し 10 秒以上持続、並びに SpO_2 の 3%以上低下を伴った場合に低呼吸と判定した。LS-140 の解析においては、体動や呼吸波形の乱れ、呼吸数の変動などから明らかな覚醒を判定し、あらかじめ記録時間より削除したものを TRT として用いた。

Statistical Analysis

主要評価項目として、PSG と LS140 の測定値を級内相関係数(Intraclass correlation coefficient: ICC)の Case2、ICC(2.1)を用いて比較した。信頼係数の定性的な評価は Landis の基準に則った。

副次評価項目として、PSG と LS140 の測定値を対応ありの T 検定によって比較した。

また、PSG の AHI と LS140 の呼吸イベント指数(respiratory event index: REI)を Bland-Altman 分析を用いて評価した。並びに AHI5 回/時間、15 回/時間、30 回/時間を診断基準とした REI の receiver operating characteristic curve (ROC 曲線)、感度と特異度を算出し比較した。また、周期性四肢運動指数(Periodic Limb Movement index: PLM Index)が 15 回/時間以上の PLMs が見られた群と見られなかった群で、それぞれ PSG と LS140 の測定値の ICC、AHI5 回/時間、15 回/時間、30 回/時間を診断基準とした REI の感度と特異度を検討した。

統計解析はパーソナル・コンピュータ用ソフトウェア「JMP ver 14 (日本語版)」(SAS Institute Inc, 東京)、と SPSS Statistics 21」(Stats Guild Inc, 千葉)を用いた。

本研究は「簡易モニターLS-140 と終夜睡眠ポリグラフとの比較研究（承認番号 HM18-048)」として、藤田医科大学医学研究倫理審査委員会の承認を得た。

結果

患者背景(表 1): 全対象者 58 名(男性 48 名、女性 10 名)の年齢中央値(四分位)は 54(43.8-63)歳、BMI 中央値(四分位)は 25(22.9-29.2)kg/m^2、AHI 中央値(四分位)は 29.1(20-42.4)回/h であり、正常(AHI<5 回/時間)3 名、軽症者(5≦AHI<15 回/時間)6 名、中等症者(15≦AHI<30 回/時間)22 名、重症者(AHI≧30 回/時間)27 名であった。うち 18 名に PLM Index が 15 回/時間以上の PLMs を認めた。対象者 58 名中のうち 1 名に LS140 の酸素飽和度センサーの脱落があり、データの欠損を生じていた。

主要評価項目: PSG と LS140 の測定値の比較(表 2)

PSG 上の AHI と LS140 による REI の ICC(2.1)は 0.944(almost perfect)と高値を示した(p<0.0001)。無呼吸指数(Apnea Index: AI)、混合性無呼吸指数(Mixed Apnea Index: MAI)、3%酸素飽和度低下指数(3% Oxygen Desaturation Index: 3%ODI)に 0.61 以上(substantial)の信頼係数(p<0.0001)を認めた。中枢性無呼吸指数(Central Apnea Index: CAI)、無呼吸最大持続時間には有意な相関を認めなかった。PSG における全睡眠時間と LS140 上の TRT の相関は 0.341 と fair に留まった。

副次評価項目: LS140 で AI、OA、3%ODI、% Time of SpO_2<90%は有意に高く、HI、REI、$LowestO_2$ は有意に低かった(表 1)。REI に関する Bland-Altman 分析では、AHI を基準とした両者の差の平均値は-3.6(p<0.0001)、95%信頼区間は-5.34～-1.93(一致限界 3.41)であり、REI は AHI に対し負の固定誤差を示すが、両者の値は近似してい

た。AI は PSG を基準にした差の平均値は 7.62(p<0.0001)、95%信頼区間は 5.53～9.71(一致限界 4.18)であり、LS140 の AI は PSG の AI に対し正の固定誤差を認めた。また、OA では PSG を基準にした差の平均値は 8.97(p<0.0001)、95%信頼区間は 6.91～11.05(一致限界 4.14)であり、LS140 の OA は PSG の OA に対し正の固定誤差を認めた。(図 1)

ROC 曲線を用いて REI による AHI の診断能を算出した(表 3)。ROC 曲線下の領域は、AHI5 回/時間以上を陽性とすると 1.000、AHI15 回/時間以上を陽性とすると 0.986、AHI30 以上で 0.962 といずれも極めて高かった。AHI5 回/時間以上を基準とした REI の感度は 0.964、特異度は 1.000、AHI30 回/時間以上を基準とすると REI の感度は 0.741、特異度は 1.000 であった。

PLMs 群 18 名と非 PLMs 群 40 名の患者背景を表 4 に示した。PLMs 群 18 名(男性 16 名、女性 2 名)は非 PLMs 群に比べ年齢が高く(p=0.011) 、PSG における酸素飽和度最低値が高い傾向が見られた(p=0.04)。AHI に有意差は見られなかった(p=0.094)。LS140 の計測値でも酸素飽和度最低値は高く(p=0.024)、3%ODI が有意に低かった(p=0.009)。

非 PLMs 群における AHI と REI の ICC(2.1)は 0.945(p<0.0001)、AI の ICC(2.1)は 0.911(p、0.0001)といずれも高値(almost perfect)を示した。しかし TST と LS140 上の TRT の ICC(2.1)は 0.471(p=0.001)と信頼係数は moderate に留まった。PLMs 群における AHI と REI の ICC(2.1)は 0.927 と almost perfect であったが、AI の ICC(2.1)は 0.233(p=0.168)と有意な相関を認めず、また、TST と LS140 上の TRT にも有意な相関を認めなかった(表 5)。

PLMs 群における ROC 曲線化の領域は、AHI5 回/時間以上を陽性とすると 1.000、AHI15 回/時間以上を陽性とすると 0.946、AHI30 回/時間以上で 0.961 といずれも高

値であった。AHI5 回/時間以上を基準とした REI の感度は 0.938、特異度は 1.000、AHI30 回/時間以上を基準とした REI の感度は 0.429、特異度は 0.909 であった(表 6)。非 PLMs 群における ROC 曲線下の領域は、AHI5 回/時間以上を陽性とすると 1.000、AHI15 回/時間以上を陽性とすると 1.000、AHI30 回/時間以上で 0.976 といずれも高値であった。AHI5 回/時間以上を基準とした REI の感度は 0.974、特異度は 1.000、AHI30 回/時間以上を基準とした REI の感度は 0.948、特異度は 1.000 であった。

考察

PMs 機器の利用増加に伴い、正確性や有用性に関する検討は多くなされてきた[14-16]。2014 年までに報告された PSG とタイプ 3 PMs の比較試験のメタ解析では、タイプ 3 PMs(気流もしくは呼吸運動、心拍数または心電図、酸素飽和度を含む最小チャンネルを記録できる機器)は、検査前確率が高い成人を対象とし、監視下で測定し専門の技師がスコアリングマニュアル(AASMver2.5)に沿って解析したデータには十分な信頼性があるとされている。しかし実際の運用において測定されるチャンネル数は施設によって異なり、チャンネル 4 での使用が多く 69.5%、チャンネル 3 での使用が 10.1%であった[17]。本研究で用いた LS-140 は圧センサーにより呼吸気流、マスク圧、気管音を、圧電センサー並びに加速度センサーにより呼吸努力と体位情報及び体動情報を、パルスオキシメーターによる SpO_2 と脈拍数を記録可能である。今回は呼吸気流、呼吸努力、体位、SpO_2、脈拍の 5 チャンネルを測定した。対象者 58 名の PSG によって判定された重症度は中等症以上が 84%を占めていた。外来での問診により睡眠時無呼吸症候群が疑われ PSG 検査を施行しており、検査前確率は一般人口よりも高いと考えられる。チェーン・ストークス呼吸含む中枢性無呼吸を示した患者は見られず、閉塞性優位の無呼吸、低呼吸が主であった。

PSG 上の AHI と LS140 による REI の ICC(2.1)は 0.944(almost perfect)と高値を示した(p<0.0001) (表 2)。2014 年に行われたランダム化クロスオーバー試験[17]における PSG と施設内測定の PMs での ICC は 0.79(95%信頼区間 0.67～0.86)であり、それを上回る結果となった。

本研究では呼吸イベントの級内相関は高値となったが、PSG における全睡眠時間(Total sleep time: TST)と LS140 上の TRT の級内相関は 0.341(fair)に留まった。TST と TRT の平均の差には有意差が見られないことが(表 1)、REI と AHI の高い相関に寄与していると思われる。PMs は臨床での運用上、機器の総記録時間から体動や呼吸の乱れなど、技師から見て明らかな覚醒時間を測定時間から除外して評価することにより PSG との近似性を改善する[10]とされているが、本研究の TST と TRT の級内相関係数が低いことから二者の値にはばらつきが大きく、検者間信頼性には課題がある。また、LS140 では PSG に比べ AI、OA に正の固定誤差を認めた(図 1)。本研究では測定時に患者に装着した鼻圧カニューレを二分岐し、PSG と LS140 の双方で等しい気圧を元に解析している。AI と OA が一定の固定誤差を持つことは LS140 の機器特性と考えられた。

ROC 曲線下の領域は、AHI5 回/時間以上、AHI15 回/時間以上、AHI30 回/時間以上のどの基準を用いても 0.96 以上の高い診断能が示された。AHI に対する REI の感度は各診断基準で 74.1～96.4%、特異度はいずれも 100%であった。先行するメタ解析[17]における検査室施行の PSG での AHI5 回/時間以上を基準とした PMs の感度の平均値(95%信頼区間)は 97(92-99)%、特異度の中央値(四分位)は 93(89-96)%あり、感度はほぼ同等、特異度は高い結果となった。

先行研究にて、PMs では四肢運動を評価できず、したがって周期性四肢運動障害による睡眠の分断を評価できないと指摘されている[18]。今回の全対象者の PLM index（回

/時）の中央値(四分位)は 1(0-28.7)、58 名中 18 名(31%)に PLM Index 15 回/時間以上の周期性四肢運動を認めた。Wisconsin Sleep Cohort study では一般人口(中央値 48 歳)の PLM-Index15 回/時間以上の有病率は 25.3%、加齢によって増加し 34%ほどに達する報告されている[19.20]。本研究の対象者の年齢層は 54(43.8-63)歳ほどであり、先行研究に矛盾しない。

PLMs 群では AHI30 回/時間をカットオフとした場合の感度が 42.9%と大幅に低下していた。脳波を欠く PMs による呼吸イベントの過小評価の程度は中途覚醒の増加に伴って悪化するため[8]、PLMs 群において感度が低下した可能性がある。同様に、LS140 によって得られた REI の陽性尤度比はいずれの診断基準対しても∞であるが、各診断基準に対する陰性尤度比は 0.259~0.555 とメタ解析での平均値 0.03(0.01-0.08)に比べやや劣る。全対象者における陰性反応的中率は 81.6%だが、非 PLMs 群では 90.9%、PLMs 群では 73.3%と差が見られた。PLMs 群の成績が全体に影響を及ぼしたものと考えられ、PMs 機器による重症者の除外には慎重を要する。

Bland-Altman 分析を用いた AHI と REI の差の平均値は-3.6(p<0.0001)と負の固定誤差を示しており(図 1)、PMs が PSG の値に比べ呼吸イベントを過小評価する一般的な傾向を反映していると考えられる。一致限界は 3.4 と両者の値は近似しており、PLMs の有無によって大きな差はみられなかった。散布図は右に向かって扇形を示しており(図 2)、PMs での数値が PSG に対し、測定値が大きくなるほど誤差を生ずることを示唆している。PLMs 群では AHI30 回/時間以上になると感度が低下するため、その影響を受けた可能性がある。REI の AHI に対する感度が AHI5 回/時間を基準とした時最も高く(96.4%)、AHI30 回/時間を基準とした時に 74.1%まで低下するのは、この誤差の広がりを反映していると考えられる。REI と AHI の差に対する AHI の相関を求めた結果、相関係数は-0.2390(95%信頼区間-0.04684～0.0205)、p 値 0.0708

と有意な相関を認めなかった(補足資料)。PLMs の有無による PMs パラメータへの影響はまだ明らかになっておらず、一般的な傾向は不明である。

Limitation

本研究においては機器の装着を技師が行っており、本来患者自身が在宅で行う場合と条件が異なる。また、二重盲検試験ではないため観察者バイアスを除去できず、外的妥当性に限界がある。

結論

PSG における AHI と LS140 による REI の相関は非常に高く、呼吸イベントの診断において十分な診断感度と特異度を有する。ただし、PLMs を有する患者は PMs での計測に大きく影響する可能性があり、一般人口の有病率が 25%と高いことからも注意を要する。LS140 のセンサーは鼻圧センサーと指先型の酸素飽和度センサー、並びにベルト型の体位センサーの三本のみであり、技師による説明とイラスト入りの説明書の利用で被験者による装着は容易である。本研究では就寝後の脱落は 58 件中 1 件のみであった。以上のことから、LS140 は在宅において非監視下で使用する PMs として OSA のスクリーニングに十分な機能を有すると結論する。

利益相反

本研究に当たって、フクダ電子株式会社より LS140 機器の貸与を受けた。

謝辞

本研究の統計手法と解析に多大なご支援を頂いた高橋宏博士に深く御礼申し上げます。査読の先生方には数々の有益なご助言を賜り、心より感謝いたします。この研究はフクダ電子株式会社より LS1407 の貸与を受けて遂行されたものです。

参考文献

1. Marin JM, Carrizo SJ, Vicente E, Agusti AG. Long-term cardiovascular outcomes in men with obstructive sleep apnoea-hypopnoea with or without treatment with continuous positive airway pressure: an observational study. Lancet 2005;365:1046-53.

2. Ulfberg J, Carter N, Talbäck M, Edling C. Excessive daytime sleepiness at work and subjective work performance in the general population and among heavy snorers and patients with obstructive sleep apnea. Chest 1996;110:659-63.

3. Tanigawa T, Tachibana N, Yamagishi K, Muraki I, Kudo M, Ohira T, Kitamura A, Sato S, Shimamoto T, Iso H. Relationship between sleep-disordered breathing and blood pressure levels in community-based samples of Japanese men. Hypertens Res 2004; 27:479-84

4. Cui R, Tanigawa T, Sakurai S, Yamagishi K, Imano H, Ohira T, Kitamura A, Sato S, Shimamoto T, Iso H. Associations of sleep-disordered breathing with excessive daytime sleepiness and blood pressure in Japanese women. Hypertens Res 2008; 31: 501-6.

5. Benjafield AV, et al. Estimation of the global prevalence and barden of obstructive sleep apnoea : a literature-based analysis. Lancet Respir Med 2019; 7 :687-98.7

6. Ministry of Health, Labour and Welfare of Japan. 2018 Statistics by medical practice. https://www.e-stat.go.jp/stat-search/file-download?statInfId=000031833867&fileKind=1. (Accessed June 22, 2020)

7. Satou M. Epidemiology of sleep apnea (In Japanese). The Journal of the Japanese Society of Internal Medicine 2020; 109 :1059-64.

8. Bianchi TM, Goparaju B. Potential underestimation of sleep apnea severity by at-home kits: rescoring in-laboratory polysomnography without sleep staging. J Clin Sleep Med 2017; 13: 551–5.

9. Nancy A. Collop, M.D, W.McDowell Anderson M.D. Brian Boehlecke, M.D.et al.Clinical Guidelines for the Use of Unattended Portable Monitors in the Diagnosis of Obstructive Sleep Apnea in Adult Patients. Journal of Clinical Sleep

Medicine; 2007 :3 :737-47

10. Nakano H. Kani monita no pittofuoru(Pitfall of home sleep testing). Suiminiryou 2011; 5: 189-93 (In Japanese).

11. Brown DL, Chervin RD, Hegeman G 3rd, Smith MA, Garcia NM, Morgenstern LB, Lisabeth LD. Is technologist review of raw data necessary after home studies for sleep apnea? J Clin Sleep Med 2014; 10: 371–75.

12. American academy of sleep medicine. The AASM manual for the scoring of sleep and associated events. version 2.5. Tokyo: Life Science; 2018: 76-89 (in Japanese).

13. Zhao YY, Weng J, Mobley DR, Wang R, Kwon Y, Zee PC, Lutsey PL, Redline S. Effect of manual editing of total recording time: implications for home sleep apnea testing. J Clin Sleep Med 2017; 13: 121–6.

14. Chesson AL Jr, Berry RB, Pack A. Practice parameters for the use of portable monitoring devices in the investigation of suspected obstructive sleep apnea in adults. Sleep 2003; 26: 907-13.

15. Andrade L, Paiva T. Ambulatory Versus Laboratory Polysomnography in Obstructive Sleep Apnea: Comparative Assessment of Quality, Clinical Efficacy,

Treatment Compliance, and Quality of Life. J Clin Sleep Med 2018; 14: 1323-31.

16. Rosenberg R, Hirshkowitz M, Rapoport DM, Kryger M. The role of home sleep testing for evaluation of patients with excessive daytime sleepiness: focus on obstructive sleep apnea and narcolepsy. Sleep Med 2019; 56: 80-9.

17. El Shayeb M, Topfer LA, Stafinski T, Pawluk L, Menon D. Diagnostic accuracy of level 3 portable sleep tests versus level 1 polysomnography for sleep-disordered breathing: a systematic review and meta-analysis. CMAJ 2014; 186: E25-51.

18. Setty AR. Underestimation of Sleep Apnea With Home Sleep Apnea Testing Compared to In-Laboratory Sleep Testing. J Clin Sleep Med 2017; 13: 531–2.

19. American Academy of Sleep Medicine. The international classification of sleep disorders. 2nd ed. Westchester, IL: American Academy of Sleep Medicine; 2005.

20. Leary EB, Moore HE 4th, Schneider LD, Finn LA, Peppard PE, Mignot E. Periodic limb movements in sleep: Prevalence and associated sleepiness in the Wisconsin Sleep Cohort. Clin Neurophysiol 2018; 129: 2306-14.

Figure legend

FIgure 1

Brand-Altman limits of agreement and mean differences between three PSG

variables and REI of LS-140

a: PSG variable: AHI

mean difference: -3.6(p<0.0001), The 95% confidence interval -5.34～-1.93, Limits of Agreement: 3.41

b: PSG variable: AI

mean difference: 7.62(p<0.0001)、The 95% confidence interval: 5.53～9.71, Limits of Agreement: 4.18

c: PSG variable: OA

mean difference: 8.98(p<0.0001)、The 95% confidence interval: 6.91～11.05, Limits of Agreement: 4.14
